# A 10-year Retrospective Review of Patient-to-Patient Transmitted Pathogens in Culture-Positive Burn Wounds at a Tertiary Burn Center

**DOI:** 10.1177/22925503241249760

**Published:** 2024-04-28

**Authors:** Patrick Jinhyung Kim, Lucas Gallo, Jeffrey Chen, Morgan Yuan, Matteo Gallo, Cheryl Main, Christopher Coroneos

**Affiliations:** 1 Division of Plastic Surgery, Department of Surgery, 3710 McMaster University, Hamilton, Canada; 2 Department of Medicine, 3710 McMaster University, Hamilton, Canada; 3 Division of Plastic and Reconstructive Surgery, Department of Surgery, University of Toronto, Toronto, Canada; 4 Division of Infectious Diseases, Department of Medicine, McMaster University, Hamilton, Canada

**Keywords:** brûlures, infection, infection nosocomiale, analyse rétrospective, burns, infection, hospital acquired infection, retrospective review

## Abstract

**Introduction:** Burn wound infection can progress to sepsis and is a significant source of morbidity and mortality. Prevalence of multidrug-resistant organisms are high in burn patients; these organisms can be transmitted between patients leading to poor outcomes. **Objectives:** To characterize patient-to-patient transmission of pathogens causing burn wound colonization at a single tertiary hospital burn center in Hamilton, Canada from 2011 to 2020. **Methods:** Retrospective chart review of patients admitted to the burn trauma unit at Hamilton General Hospital between 2011 and 2020. Antibiotic susceptibility panels of pathogens cultured from burn patients’ wound swab/tissue cultures were compared against pathogens cultured from other burn/nonburn patients with overlapping admission dates. Pathogens were categorized into likely, possible, or unlikely transmission, or normal skin flora on a case-by-case basis. **Results:** There were 173 burn patients with positive wound culture and 613 nonburn patients included in the study. Included burn patients had median age 52 years, mostly male (73%) with flame injury (65%), and median total body surface area 18%. There were 18 patients (10%) with likely transmission and 54 patients (31%) with possible transmission. Most frequently implicated pathogens for likely patient-to-patient transmission were methicillin-resistant *Staphylococcus aureus* (MRSA) (7 patients) and methicillin-resistant coagulase-negative Staphylococci (4 patients). Both burn and nonburn patients were implicated. **Conclusion:** The burden of patient-to-patient transmission in culture-positive burn wounds was estimated to be between 10% and 41%. Greater care should be taken to avoid patient-to-patient transmission of pathogens to minimize burn infection morbidity and mortality. Prospective studies should be conducted with genomic sequencing and correlation with clinical outcomes.

## Résumé

**Introduction:** L’infection des plaies par brûlure, qui peut évoluer en sepsis, est une source importante de morbidité et de mortalité. La prévalence d’organismes pharmacorésistants est élevée chez les patients brûlés; ces organismes peuvent être transmis entre patients et entraîner de mauvais résultats cliniques. **Objectifs:** Caractériser la transmission d’agents pathogènes entre patients, responsable de la colonisation de plaies par brûlure dans un centre hospitalier de soins tertiaires pour brûlés de Hamilton, au Canada, entre 2011 et 2020. **Méthodologie:** Les chercheurs ont procédé à l’analyse rétrospective des dossiers de patients admis à l’unité des brûlés du Hamilton General Hospital entre 2011 et 2020. Ils ont comparé des panneaux de sensibilité antibiotique d’agents pathogènes prélevés sur des tissus ou des écouvillons de plaies par brûlure et mis en culture à des agents pathogènes également mis en culture, mais prélevés chez d’autres patients brûlés ou non brûlés dont les dates d’hospitalisation se recoupaient. En fonction de chaque cas, ils ont réparti les agents pathogènes entre une transmission probable, possible ou peu probable ou une flore cutanée normale. **Résultats:** Les résultats des cultures de plaie de 173 patients brûlés étaient positifs, de même que ceux de 613 patients non brûlés inclus dans l’étude. Les patients brûlés avaient un âge médian de 52 ans, étaient surtout de sexe masculin (73%) et avaient des blessures par flamme (65%), sur une surface corporelle totale médiane de 18%. Ainsi, 18 patients (10%) présentaient une transmission probable et 54 (31%), une transmission possible. Le SARM (sept patients) et les staphylocoques à coagulase négative résistant à la méthicilline (quatre patients) étaient les agents pathogènes les plus responsables d’une transmission entre patients. Tant des patients brûlés que non brûlés étaient touchés. **Conclusion:** Le fardeau de la transmission entre patients ayant une plaie par brûlure positive à la culture était estimé entre 10% et 41%. Il faudrait prendre plus de précautions pour éviter la transmission des agents pathogènes entre patients afin de réduire la morbidité et la mortalité découlant des infections par brûlure. Il faudrait réaliser des études prospectives avec séquençage génomique et les corréler avec les résultats cliniques.

## Introduction

Burn injuries are a significant source of morbidity and mortality, burdening healthcare systems. From 2009 to 2018, the American Burn Association National Burn Repository (ABA-NBR) amalgamated data from 221,519 acute burn admissions at 101 North American hospitals to measure the burden of burns.^1^ Burn patients are hospitalized on average for 8.5 to 10 days with a mortality rate of ∼3%.^1^ Among more severe burns affecting greater total body surface area (TBSA), mortality has been found to be as high as 30%. Burns also bear significant economic burden in the healthcare system; burn survivors incurred average hospital charges of $98,000 while nonsurvivors were charged $310,000.^1^

Wound infection, the fifth most common complication of burns, further compounds the complexity of burn care.^1^ The typical sequela of burn wound infection begins with a wound sterilized by the initial thermal insult, followed by colonization. When burn wound colonization is left untreated, it can progress to wound infection and eventually sepsis.^2^ The mortality rate of burn patients with infection is twice that of those without infection.^3,4^ This is primarily due to the progression to sepsis which is responsible for 42% to 65% of deaths from burn injury.^5–8^ In patients with severe burns (>40% TBSA), sepsis from burn wound infection and/or inhalation injury caused 75% of the deaths.^9^ Multidrug-resistant organisms (MDRO) such as *Pseudomonas* spp., *Acinetobacter baumannii*, *Stenotrophomonas maltophilia*, and *Staphylococcus aureus* exacerbates this health crisis.^10^ Alarmingly, another Canadian study found that the proportion of multidrug-resistant bacteria species isolated from burn infections increased from 6% at 1 week to 44% after 28 days of hospitalization.^11^

Healthcare-associated infections (HAI) are the most common type of burn wound infection; previously identified risk factors include patient factors (age and comorbidities) and burn severity (TBSA, burn depth, and inhalational injury).^4,12,13^ Sources of infection can be endogenous, meaning from the patient's own skin, mucosal surfaces, or bodily fluids, or exogenous, meaning from hospital equipment, staff, and other people.^14^ With regard to patient-to-patient transmission, the hospital environment contains numerous surfaces that act as reservoirs for pathogenic organisms. Even without direct patient contact, healthcare workers can expose immunocompromised patients (ie, burn patients) to exogenous pathogens they would otherwise not have encountered, leading to colonization then infection. Therefore, burn centers must implement strict infection prevention and control measures to minimize HAI across the entire continuum of care.^15^

To our knowledge, there have been no studies to date that investigated patient-to-patient transmission of burn wound pathogens. The primary objective of this study is to estimate the burden of patient-to-patient transmission of pathogens causing burn wound colonization at a single tertiary hospital burn center in Hamilton, Canada from 2011 to 2020. Our secondary objective was to describe the important implicated pathogens and their possible transmission sources.

## Methods

### Study Design

This was a retrospective observational chart review performed on all patients admitted to the burn trauma unit (BTU) between 2011 and 2020 at the Hamilton General Hospital (HGH) in Hamilton, Canada. The HGH is a tertiary burn center with a catchment area of approximately 7 million people. Patient data was collected from electronic medical records from a single network.

### Research Ethics

This study was approved by the Hamilton integrated Research Ethics Board (HiREB), application #13489.

### Patient Identification

All patients admitted to the BTU at HGH between 2011 and 2020 were identified. Of these patients, patients with a burn diagnosis were identified by ICD-10 diagnosis codes produced with provincial burn reporting data, and the 2 groups were divided into culture-positive burn patient and nonburn patient groups. At our institution, burn wound cultures are collected when indicated by signs of clinical wound infection. Criteria to determine burn wound infection include conversion of superficial partial-thickness to deep partial-thickness injury, change in wound/skin appearance, eschar separation, and delayed healing/graft issues.

Within the culture-positive burn patient group, patients were included if: (1) they were 18 years or older; (2) they were admitted to the BTU for ≥1 day for a primary burn diagnosis; (3) they had a positive wound swab or tissue culture during their admission. Re-admissions to the BTU following previous primary admissions were not included for analysis.

Within the nonburn patient group, patients were included if: (1) they were 18 years or older; (2) they were admitted to the BTU for ≥1 day; (3) they had admission dates that overlapped with at ≥1 included burn patient; (4) they had a positive wound, blood, line, stool, or urine culture during their admission.

### Data Collection

Patient data was retrospectively collected via electronic medical records. Demographic and clinical information were extracted in duplicate for 20% of records using piloted extraction forms. Disagreements were resolved by consensus and input from the primary investigator as needed. The remaining 80% of data was extracted independently.

Specifically with regard to culture data, the following information was extracted: pathogen type, culture source (wound [tissue or swab], blood, lines, stool, urine), and specimen number. Specimen numbers were given to the infectious disease specialist on the research team, who then collected susceptibility panel data from the hospital laboratory database.

### Outcomes

The primary outcome of this study was the proportion of culture-positive burn patients likely or possibly implicated in patient-to-patient transmission of pathogens with another patient in the BTU. For each pathogen identified on positive culture from the culture-positive burn patient group, susceptibility data were compared with susceptibility panel data for both burn and nonburn patients meeting the following criteria: (1) the same pathogen identified on positive culture and (2) overlapping dates of admission. These patients were defined as the “comparator” group.

Each pathogen for each culture-positive burn patient was appraised with the help of an academic infectious disease specialist on our research team. The pathogens were categorized into likely, possible, or unlikely transmission, or normal skin flora. Although all decisions were made on a case-by-case basis for each pathogen and susceptibility data, the general appraisal went as follows:

- Likely transmission: pathogens with identical susceptibility panels with notable antibiotic resistance(s);
- Possible transmission: pathogens with identical but typical susceptibility panels or nonidentical susceptibility panels with similar notable antibiotic resistance(s);
- Unlikely transmission: pathogens with differing susceptibility panels with key differences in antibiotic resistance;
- Normal skin flora: pathogens found as part of normal skin flora without notable antibiotic resistance(s);
- No overlap: pathogens and admission dates did not overlap with other patients in the BTU.

As additional secondary outcomes, we reported which pathogens were implicated in likely and possible patient-to-patient transmissions.

### Data Analysis

Descriptive statistics were used to report patient and pathogen characteristics and the primary outcome (proportion of culture-positive burn patients likely implicated in patient-to-patient transmission of pathogens with another patient in the BTU). To compare proportion of culture-positive wounds with likely patient-to-patient transmission before and after new infection control measures, a Chi-square test was used. Statistical significance was determined to be *P* < .05.

## Results

**Figure 1.**
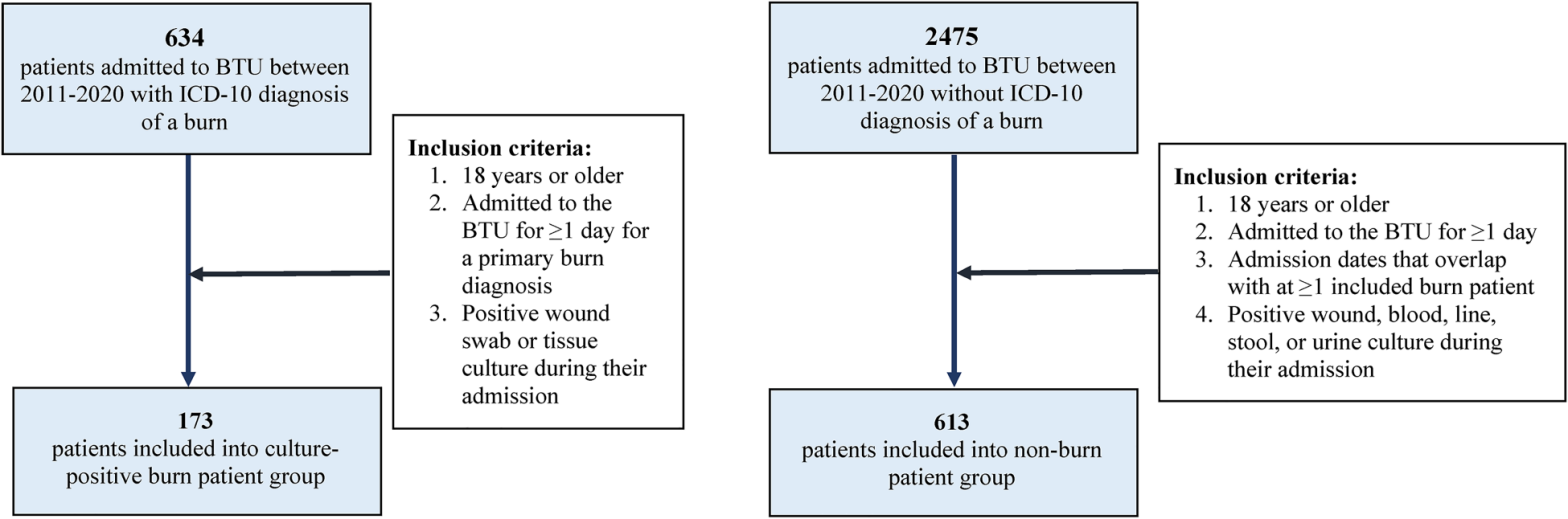
Inclusion flowchart for colonized burn patient and nonburn patient groups. Abbreviation: BTU, burn trauma unit.

Of the 634 patients admitted to the BTU between 2011 and 2020 with an ICD-10 diagnosis of a burn, there were 173 patients who met inclusion criteria for the culture-positive burn patient group. Of the 2475 patients admitted to the BTU between 2011 and 2020 without ICD-10 diagnosis of a burn, there were 613 patients who met inclusion criteria for the nonburn patient group (Figure 1).

Included burn patients had median age 52 years (interquartile range [IQR]: 40-62), mostly male (73%) with flame injury (65%), and median total body surface area was 18%. Most patients had burns on their upper limb (75%) and trunk/abdomen (62%). Inhalational injury was present in 19% of patients and 54% had full-thickness burns. Table 1 shows demographic data in more detail.

**Table 1.** Baseline Characteristics of Included Culture-Positive Burn Patients.

Patient characteristic	n/N (%) or median (IQR)
Age (years)	52 (40-62)
*18-40*	45/173
*41-60*	79/173
*61-80*	39/173
*81+*	10/173
Sex	
*Male*	126/173 (
*Female*	47/173 (
Smoking status	
*Current*	66/173
*Former*	14/173
*Never*	53/173
*NR*	39/173
Diabetic	27/173
Burn TBSA	18 (7-37.5)
*0-9%*	51/173
*10-19%*	36/173
*20-29%*	28/173
*30+%*	47/173
*NR*	9/173
Burn site	
*Head and neck*	70/173
*Upper limb*	130/173
*Lower limb*	93/173
*Trunk/abdomen*	107/173
Inhalation injury	32/173
Full thickness burn	94/173
Burn etiology^+^	
*Flame*	113/173
*Contact*	23/173
*Scald*	20/173
*Chemical*	18/173
*Electrical*	8/173
*NR*	4/173

+ One patient had both a flame and a contact burn.

Abbreviations: IQR, interquartile range; NR, not reported; TBSA, total body surface area.

With regard to likelihood of patient-to-patient transmission of pathogens, 10% (n = 18/173) of included burn patients had at least one likely transmission of pathogens. There were 31% (n = 54/173) with at least one possible transmission and 17% (n = 30/173) with at least one unlikely transmission. The remaining 25% (n = 44/173) only had normal skin flora and 33% (n = 57/173) had no overlapping pathogens/admission dates with other patients.

### Likely Transmission

Among the 18 patients with likely transmission of pathogens, there were 19 events that were identified (Table 2). Overall, nearly all (n = 18/19) likely transmission events occurred between 2015 and 2020, with a single event in 2012. Burn patient wound culture sources were a combination of wound swabs (47%, n = 9/19), tissue cultures (21%, n = 4/19), and both wound swab and tissue cultures (32%, n = 6/19). Comparator patient culture sources were either from wounds (42%, n = 8/19), blood (11%, n = 2/19), or multiple sources (37%, n = 9/19). Patients in the comparator group consisted of other burn patients (42%, n = 8/19), nonburn patients (21%, n = 4/19), and a combination of both burn and nonburn patients (37%, n = 7/19).

**Table 2.** Overview of Likely Transmission Events.

Patient	Pathogen	Year	Culture-positive burn patient		Comparator		
			Specimen source	Susceptibility profile	Specimen source(s)	Susceptibility profile	Patient type
1	*Acinetobacter baumannii complex*	2020	Tissue culture	Sensitive to: Ceftazidime Tobramycin TMP-SMX Meropenem Resistant to: Cefazolin Ciprofloxacin Indeterminate to: Ceftriaxone Pip-Tazo	Blood	Sensitive to: Ceftazidime Gentamicin Tobramycin TMP-SMX Ceftriaxone Pip-Tazo Meropenem Resistant to: Cefazolin Ciprofloxacin	Burn
2	*Pseudomonas aeruginosa*	2020	Wound swab/tissue culture	Sensitive to: Ceftazidime Gentamicin Tobramycin Pip-Tazo Resistant to: Meropenem Indeterminate to: Ciprofloxacin	Wound swab/tissueculture/ urine	Sensitive to: Ceftazidime Gentamicin Tobramycin Pip-Tazo Resistant to: Meropenem Indeterminate to: Ciprofloxacin	Burn
3	*Acinetobacter baumannii complex*	2020	Tissue culture	Sensitive to: Ceftazidime Gentamicin Tobramycin TMP-SMX Ceftriaxone Pip-Tazo Meropenem Resistant to: Cefazolin Indeterminate to: Ciprofloxacin	Blood	Sensitive to: Ceftazidime Tobramycin TMP-SMX Ceftriaxone Pip-Tazo Meropenem Resistant to: Cefazolin Indeterminate to: Ciprofloxacin Gentamicin	Burn
					Wound swab	Sensitive to: Ciprofloxacin Gentamicin Tobramycin TMP-SMX Pip-Tazo Meropenem Resistant to: Cefazolin Indeterminate to: Ceftazidime	Nonburn
4	*P. aeruginosa*	2020	Wound swab/ tissue culture	Sensitive to: Ceftazidime Gentamicin Tobramycin Pip-Tazo Resistant to: Meropenem Indeterminate to: Ciprofloxacin	Wound swab/ tissue culture	Sensitive to: Ceftazidime Gentamicin Tobramycin Pip-Tazo Resistant to: Meropenem Indeterminate to: Ciprofloxacin	Burn
5	MRSA	2019	Wound swab	Sensitive to: Clindamycin Gentamicin Nitrofurantoin Rifampin Tetracycline TMP-SMX Vancomycin Resistant to: Cefazolin Ciprofloxacin Erythromycin Levofloxacin Cloxacillin	Wound swab (1), tissue culture (1)	Sensitive to: Clindamycin Gentamicin Nitrofurantoin Rifampin Tetracycline TMP-SMX Vancomycin Resistant to: Cefazolin Ciprofloxacin Erythromycin Levofloxacin Cloxacillin	Burn & nonburn
6	MRSA	2019	Wound swab	Sensitive to: Clindamycin Gentamicin Nitrofurantoin Rifampin Tetracycline TMP-SMX Vancomycin Resistant to: Cefazolin Ciprofloxacin Erythromycin Levofloxacin Cloxacillin	Wound swab	Sensitive to: Clindamycin Gentamicin Nitrofurantoin Rifampin Tetracycline TMP-SMX Vancomycin Resistant to: Cefazolin Ciprofloxacin Erythromycin Levofloxacin Cloxacillin	Burn
7	MRSA	2019	Wound swab	Sensitive to: Clindamycin Gentamicin Nitrofurantoin Rifampin Tetracycline TMP-SMX Vancomycin Resistant to: Cefazolin Ciprofloxacin Erythromycin Levofloxacin Cloxacillin	Wound swab (1), wound swab/tissue culture (1), blood (1)	Sensitive to: Clindamycin Gentamicin Nitrofurantoin Rifampin Tetracycline TMP-SMX Vancomycin Resistant to: Cefazolin Ciprofloxacin Erythromycin Levofloxacin Cloxacillin	Burn & nonburn
8	MRSA	2018-2019	Wound swab	Sensitive to: Clindamycin Gentamicin Nitrofurantoin Rifampin Tetracycline TMP-SMX Vancomycin Resistant to: Cefazolin Ciprofloxacin Erythromycin Levofloxacin Cloxacillin	Wound swab (3), wound swab/tissue culture (1), blood (2)	Sensitive to: Clindamycin Gentamicin Nitrofurantoin Rifampin Tetracycline TMP-SMX Vancomycin Resistant to: Cefazolin Ciprofloxacin Erythromycin Levofloxacin Cloxacillin	Burn & nonburn
9	MRSA	2018	Wound swab/ tissue culture	Sensitive to: Clindamycin Gentamicin Nitrofurantoin Rifampin Tetracycline TMP-SMX Vancomycin Resistant to: Cefazolin Ciprofloxacin Erythromycin Levofloxacin Cloxacillin	Wound swab (4), blood (2)	Sensitive to: Clindamycin Gentamicin Nitrofurantoin Rifampin Tetracycline TMP-SMX Vancomycin Resistant to: Cefazolin Ciprofloxacin Erythromycin Levofloxacin Cloxacillin	Burn & nonburn
10	MRSA	2018	Wound swab	Sensitive to: Clindamycin Gentamicin Nitrofurantoin Rifampin Tetracycline TMP-SMX Vancomycin Resistant to: Cefazolin Ciprofloxacin Erythromycin Levofloxacin Cloxacillin	Wound swab/tissue culture	Sensitive to: Clindamycin Gentamicin Nitrofurantoin Rifampin Tetracycline TMP-SMX Vancomycin Resistant to: Cefazolin Ciprofloxacin Erythromycin Levofloxacin Cloxacillin	Burn
					Blood	Sensitive to: Clindamycin Gentamicin Nitrofurantoin Rifampin Tetracycline TMP-SMX Vancomycin Resistant to: Cefazolin Ciprofloxacin Erythromycin Levofloxacin Cloxacillin Penicillin	Nonburn
11	MRSA	2018	Wound swab	Sensitive to: Clindamycin Gentamicin Nitrofurantoin Rifampin Tetracycline TMP-SMX Vancomycin Resistant to: Cefazolin Ciprofloxacin Erythromycin Levofloxacin Cloxacillin Penicillin	Wound swab	Sensitive to: Clindamycin Gentamicin Nitrofurantoin Rifampin Tetracycline TMP-SMX Vancomycin Resistant to: Cefazolin Ciprofloxacin Erythromycin Levofloxacin Cloxacillin	Nonburn
12	Coagulase-negative Staphylococci	2017	Wound swab	Sensitive to: Clindamycin Gentamicin Nitrofurantoin Rifampin Tetracycline Vancomycin Resistant to: Cefazolin Ciprofloxacin Erythromycin Levofloxacin Cloxacillin Penicillin TMP-SMX	Wound swab	Sensitive to: Clindamycin Gentamicin Nitrofurantoin Rifampin Tetracycline Vancomycin Resistant to: Cefazolin Ciprofloxacin Erythromycin Levofloxacin Cloxacillin TMP-SMX	Burn
13	Coagulase-negative Staphylococci	2017	Wound swab	Sensitive to: Clindamycin Gentamicin Nitrofurantoin Rifampin Tetracycline Vancomycin Resistant to: Cefazolin Ciprofloxacin Erythromycin Levofloxacin Cloxacillin TMP-SMX	Wound swab	Sensitive to: Clindamycin Gentamicin Nitrofurantoin Rifampin Tetracycline Vancomycin Resistant to: Cefazolin Ciprofloxacin Erythromycin Levofloxacin Cloxacillin Penicillin TMP-SMX	Burn
14	*Streptococcus mitis*	2017	Tissue culture	Sensitive to: Vancomycin Ceftriaxone Indeterminate to: Penicillin	Blood	Sensitive to: Vancomycin Ceftriaxone Indeterminate to: Penicillin	Burn
15	*Serratia marcescens*	2016-2017	Tissue culture	Sensitive to: Amikacin Ciprofloxacin Ceftazidime Gentamicin Tobramycin TMP-SMX Ceftriaxone Ertapenem Meropenem Resistant to: Ampicillin Cefazolin Nitrofurantoin	Wound swab/ tissue culture	Sensitive to: Amikacin Ciprofloxacin Cefixime Ceftazidime Gentamicin Ceftriaxone Ertapenem Meropenem Resistant to: Ampicillin Cefazolin Nitrofurantoin	Nonburn
	Vancomycin-resistant *Enterococcus faecium*		Wound swab/ tissue culture	Sensitive to: Gentamicin Nitrofurantoin Resistant to: Ciprofloxacin Levofloxacin Penicillin Tetracycline Vancomycin Ampicillin	Tissue culture/stool	Resistant to: Ciprofloxacin Levofloxacin Gentamicin Penicillin Vancomycin Ampicillin Indeterminate to: Nitrofurantoin	Nonburn
16	Coagulase-negative Staphylococci	2016	Wound swab/ tissue culture	Sensitive to: Nitrofurantoin Rifampin Vancomycin Resistant to: Cefazolin Ciprofloxacin Clindamycin Erythromycin Levofloxacin Gentamicin Imipenem Cloxacillin Penicillin Tetracycline TMP-SMX	Wound swab/ tissue culture/blood	Sensitive to: Nitrofurantoin Rifampin Vancomycin Resistant to: Cefazolin Ciprofloxacin Clindamycin Erythromycin Levofloxacin Gentamicin Imipenem Cloxacillin Penicillin Tetracycline TMP-SMX Clarithromycin Ceftriaxone Cefurox	Burn
17	Coagulase-negative Staphylococci	2015-2016	Wound swab/ tissue culture	Sensitive to: Nitrofurantoin Rifampin Vancomycin Resistant to: Cefazolin Ciprofloxacin Clindamycin Erythromycin Levofloxacin Gentamicin Imipenem Cloxacillin Penicillin Tetracycline TMP-SMX Clarithromycin Ceftriaxone Cefurox	Wound swab/ tissue culture/blood	Sensitive to: Nitrofurantoin Rifampin Vancomycin Resistant to: Cefazolin Ciprofloxacin Clindamycin Erythromycin Levofloxacin Gentamicin Imipenem Cloxacillin Penicillin Tetracycline TMP-SMX	Burn
18	*Staphylococcus aureus*	2012	Wound swab	Sensitive to: Cefazolin Ciprofloxacin Levofloxacin Gentamicin Imipenem Nitrofurantoin Cloxacillin Rifampin Tetracycline TMP-SMX Vancomycin Ceftriaxone Cefurox Resistant to: Clindamycin Erythromycin Penicillin Clarithromycin	Wound swab	Sensitive to: Cefazolin Ciprofloxacin Levofloxacin Gentamicin Imipenem Nitrofurantoin Cloxacillin Rifampin Tetracycline TMP-SMX Vancomycin Ceftriaxone Cefurox Resistant to: Clindamycin Erythromycin Penicillin Clarithromycin	Nonburn

Abbreviations: MRSA, methicillin-resistant *Staphylococcus aureus;* Pip-Tazo,Piperacillin-Tazobactam; TMP-SMX, Trimethoprim-Sulfamethoxazole.

The pathogen most commonly involved in likely transmission events was methicillin-resistant *S. aureus* (MRSA), which had 7 events; all 7 events occurred between 2018 and 2019. Next, there were 4 likely transmission events of methicillin-resistant coagulase-negative Staphylococci which occurred between 2 pairs of burn patients in 2015/2016 and 2017. Other implicated pathogens were *A. baumannii complex* (2)*, Pseudomonas aeruginosa* (2)*, Serratia marcescens, S. aureus, Streptococcus mitis,* and vancomycin-resistant *Enterococcus faecium*.

### Possible Transmission

Among the 54 patients with possible transmission of pathogens, there were 80 events identified. The most common pathogens were *S. aureus* (16), *P. aeruginosa* (13), and *Enterococcus faecalis* (12). Table 3 summarizes all pathogens identified in possible transmission events.

**Table 3.** Summary of Pathogens Identified in Possible Transmission Events.

Pathogen	N
*Escherichia coli*	4
*Enterobacter cloacae* complex	3
*Enterococcus cloacae* complex	1
*Enterococcus faecalis*	12
*Enterococcus gallinarum*	2
*Enterococcus* species	6
*Klebsiella pneumonia*	1
MRSA	4
Multidrug-resistant *Pseudomonas aeruginosa*	2
*P. aeruginosa*	13
*Serratia marcescens*	1
*Staphylococcus aureus*	16
*Staphylococcus epidermidis*	3
*Staphylococcus haemolyticus*	7
*Staphylococcus lugdunensis*	2
*Stenotrophomonas maltophilia*	1
*Streptococcus agalactiae Group B*	1
*Streptococcus pyogenes Group A*	1
Total	80

Abbreviation: MRSA, methicillin-resistant *Staphylococcus aureus.*

## Discussion

The present study investigated culture-positive burn wounds at a tertiary burn center in Hamilton, Canada between 2010 and 2020 and found that among 173 patients with culture-positive burn wounds, 10% were likely to be involved in patient-to-patient transmission, and 31% were possibly involved in patient-to-patient transmission. Thus, the burden of patient-to-patient transmission of pathogens among culture-positive burn wounds is estimated to be 10% to 41%. If the above is extended to all 634 patients admitted to the BTU between 2010 and 2020, the estimated incidence of patient-to-patient transmission of pathogens is 3% to 12% overall. The remaining 59% to 90% of culture-positive burn wounds may be caused by both endogenous and exogenous sources and may or may not be healthcare associated.

Prior to hospital admission, many patients may introduce contamination to the burn wound leading to early burn wound cellulitis, which is not an HAI. A retrospective study in the United States showed a 16.3% incidence of burn wound cellulitis among patients admitted for a burn.^16^ Causes for burn wound cellulitis can be due to events immediately following the burn such as the thermal source itself, rolling on the ground, application of cool liquids/surfaces, etc.^16^

In the hospital environment, burn patients are at significant risk of acquiring HAIs. HAI in burn patients have been described in the past both by other institutions around the world^4,17,18^ as well as our own,^13^ and the incidence of HAI is estimated to be between 16% and 28%. Risk factors for HAI have been shown to be older age, underlying comorbidities, increasing TBSA, increasing burn depth, inhalation injury, among others.^4,13,17^ Common pathogens implicated in burn wound HAIs are coagulase-negative Staphylococci, *S. aureus, P. aeruginosa,* and *A. baumannii*, with some variations between studies.^4,13,17,18^

The distinction between the present study and previous investigations of HAI in burn patient populations is that the present study aimed to look specifically at patient-to-patient transmission, which is one of the ways patients can acquire an HAI. Pathogens from one patient can be transmitted to another via healthcare workers, hospital surfaces/equipment, or direct patient contact.^14^ Patient-to-patient transmission is of particular importance in the burn patient population, where a combination of decreased immune function, large open wounds, long hospital stays, and prolonged antibiotic courses create ideal conditions for MDROs.^19^ Unsurprisingly, MDROs have been found to be more common in burn patients than other patient populations.^20^

By nature of our study methodology's focus around susceptibility panels to determine likelihood of transmission, many of the isolates from likely transmission events feature MDROs. Most commonly, we identified 7 patients with likely transmission of *MRSA* and 4 patients with methicillin-resistant coagulase-negative Staphylococci*.* Other notable MDROs include a pair of burn patients with meropenem-resistant *P. aeruginosa* and a case of vancomycin-resistant *E. faecium* involved with a nonburn patient.

Notably, in our study, the patients in the comparator group implicated in likely transmission consisted of both burn and nonburn patients. During the time period of study at our institution, the BTU was also utilized as the plastic surgery service inpatient ward. This population can range from finger replants to sarcoma reconstructions to necrotizing fasciitis patients. By being admitted in the BTU, these patients may have been exposed to MDROs that they otherwise would not have encountered on a typical surgical ward. This highlights the importance of appropriate infection prevention and control measures to minimize the spread of infection.

For burn wound infections, airborne, droplet, and contact spread have all been described.^21^ To reduce or prevent the spread of pathogens from patient to patient, several factors must be considered. The Public Health Agency of Canada recommends the following routine precautions for healthcare workers: hand hygiene, appropriate personal protective equipment, and minimizing patient transport. From an administrative perspective, there should be appropriate cleaning of rooms and equipment, patient/visitor/healthcare worker education, management of airflow in patient rooms, and isolation measures when necessary.^22^ In our BTU, in addition to the above, our infection prevention and control department follows positive wound and other body fluid cultures, making changes to isolation status as necessary. We also work closely with the infectious diseases team to practice good antimicrobial stewardship. The BTU director routinely monitors these practices to ensure appropriate adherence to the protocols in place.

Our study had some limitations. Firstly, due to the retrospective design, we did not have access to genetic sequencing or typing data to confirm the relatedness of the organisms cultured. Thus, the certainty of evidence for transmission of pathogens is limited by hypotheses based on their susceptibilities. Methodology for analysis of patient-to-patient transmission of pathogens has been previously described in a cohort study in the intensive care unit setting, which could be adapted for future investigations on this topic.^23^ Additionally, the methodology of the present study could not account for transmission of pathogens between patients without overlapping admission dates from reservoirs. The phenomenon of patients acquiring pathogens from patients who previously occupied the room before them is previously described in the literature.^24^

Finally, there was limited access to hospital records due to the use of paper charting during the time period being studied. The only clinical records available were admission notes, consultation notes, and discharge summaries. As a result, the positive cultures could not be reliably correlated with records of clinical infection or its management. This could be addressed in future studies either via prospective study design or circumvented completely in the age of electronic medical record systems; notably, the authors’ institution transitioned to a fully electronic medical record as of 2021.

## Conclusion

This was the first study of its kind to estimate the burden of patient-to-patient transmission of pathogens in culture-positive burn wounds. At our tertiary burn center in Hamilton, Canada between 2010 and 2020, the burden of patient-to-patient transmission is likely to be 10% and possibly up to 41%. Both burn and nonburn patients were involved. The most common pathogens that were likely involved in patient-to-patient transmission were *MRSA* and methicillin-resistant coagulase-negative Staphylococci*.* Our findings highlight important areas for improvement within our burn unit with regard to isolation, hand hygiene, and equipment/surface cleaning, etc.

Given the high risk of morbidity and mortality with burn wound infection, it is paramount that appropriate infection prevention and control measures are in place to minimize transmission of infectious agents. Future studies on this topic can build upon the methodology with a prospective study design to allow for genomic sequencing and better correlation of microbiological findings with clinical outcomes.
